# Lightweight 2D-Convolutional Neural Network for Drug-Kinase Binding Affinity Prediction with Minimal Encoded Representations

**DOI:** 10.5812/ijpr-172781

**Published:** 2026-09-26

**Authors:** Hassan Zohrevand, Saeid Afshar, Mohsen Ahmadi

**Affiliations:** 1 Department of Biomedical Engineering and Medical Physics, School of Medicine, Shahid Beheshti University of Medical Sciences Tehran Iran; 2 Cancer Research Center, Institute of Cancer, Avicenna Health Research Institute, Hamadan University of Medical Sciences Hamadan Iran; 3 Department of Medical Biotechnology, School of Advanced Medical Sciences and Technologies, Hamadan University of Medical Sciences Hamadan Iran

**Keywords:** Artificial Intelligence, Convolutional Neural Network, Binding Affinity, Label Encoding

## Abstract

**Background:**

Drug–kinase binding affinity (DKBA) prediction plays a crucial role in prioritizing compounds and reducing the time and financial resources required for drug discovery. However, many existing deep learning models rely on complex molecular representations, such as molecular graphs or pretrained embeddings, which may increase computational complexity and limit scalability.

**Objectives:**

This study developed a lightweight two-dimensional convolutional neural network (2D-CNN)-based model to predict DKBA, providing an efficient, low-cost framework for early-stage virtual screening of kinase inhibitors using label-encoded sequences. The primary objective was to determine whether a relatively simple CNN architecture could achieve useful predictive performance without complex molecular representations.

**Methods:**

Drug SMILES strings and protein amino acid sequences were label-encoded and reshaped into two-dimensional matrix formats (10 × 10 for drugs and 40 × 25 for proteins) without using graph-based representations or pretrained embeddings. The proposed 2D-CNN model was trained on the KIBA dataset containing 118,254 drug–protein interactions and evaluated using mean squared error (MSE), root mean squared error (RMSE), and the concordance index (CI). Dropout, regularization, and early stopping were applied during training.

**Results:**

The proposed model achieved acceptable predictive performance on the KIBA test set, with an MSE of 0.184, RMSE of 0.429, and CI of 0.870. The model comprised 179,649 trainable parameters and achieved an inference throughput of approximately 1,674 samples per second on the evaluation hardware. The model showed numerically better performance than the literature-reported results for SimBoost and KronRLS, and performance comparable to that reported for DeepDTA and WideDTA. However, these benchmark values were obtained from the original publications rather than reproduced under identical data-splitting and preprocessing conditions and, therefore, do not constitute strictly controlled head-to-head comparisons.

**Conclusions:**

The proposed framework demonstrates that a lightweight 2D-CNN can provide useful DKBA predictions using simple label-encoded ligand and protein sequences. This approach may serve as a foundation for computational DKBA prediction; however, further validation using independent and disease-specific datasets, improved sequence representations, and more stringent validation strategies is needed to assess its generalizability and practical utility.

## 1. Background

Predicting drug–kinase binding affinity (DKBA) is an important step in evaluating drug efficacy (1). This prediction assesses the strength of interactions between drug molecules and their target proteins (2), which often form the basis of a drug’s mechanism of action in treating disease. Binding affinity directly influences drug efficacy, and inaccurate predictions can contribute to failures in clinical trials. One major challenge in drug development is the high failure rate in phase II clinical trials (3), which frequently results from the inability of drugs to effectively inhibit their biological targets.

The drug development process typically takes more than 15 years and costs between $1 and $2 billion (4-6). A major reason for these substantial costs and long timelines is the failure of drugs in late-stage development, particularly when they fail to adequately inhibit target proteins (7, 8). These setbacks can waste substantial investments made during the research and development, preclinical, and clinical stages. Computational approaches, such as artificial intelligence (AI)-based DKBA prediction, may play an important role in reducing development time and cost (9). AI technologies may improve the identification of effective compounds, potentially increasing the success of preclinical and clinical development while reducing associated costs and timelines (10).

In some studies, DKBA is predicted using binary classification methods that indicate only the presence or absence of binding (11-14). These methods do not determine a precise DKBA value and therefore do not allow quantitative comparisons of drugs against a specific target. In contrast, regression models can estimate a continuous binding-affinity value rather than simply indicating binding. This continuous representation enables quantitative comparisons of candidate compounds and can support the prioritization of interactions for subsequent evaluation. The 2 classical methods for DKBA prediction, KronRLS (15) and SimBoost (16), rely on drug and protein similarities and hand-crafted features and have achieved good performance. However, because they depend on manually engineered features and have limited capacity to learn complex representations, more advanced approaches are still needed.

Deep learning has emerged as an important approach for DKBA prediction because neural networks can learn complex nonlinear relationships from high-dimensional biological and chemical data. With the availability of large datasets such as KIBA, several deep learning models have been proposed, including DeepDTA (17) and WideDTA (18), which use convolutional architectures to extract features directly from SMILES strings and protein sequences. These approaches have demonstrated the utility of sequence-based convolutional models for DKBA prediction, whereas more recent methods have explored richer molecular representations and more elaborate architectures.

More recent studies have explored graph-based encoders (19-21) and attention-driven architectures (22, 23). These approaches can provide richer representations and greater modeling capacity, although they may generally require more complex preprocessing and computational resources. Therefore, the present study examines whether acceptable predictive performance can be achieved using a simpler and more accessible architecture, with reduced representational and computational complexity, rather than attempting to outperform more complex approaches.

A deep learning model based on a two-dimensional convolutional neural network (2D-CNN) (24) was developed and implemented for DKBA prediction. The proposed lightweight dual-channel model focuses on kinase proteins, which play a central role in cancer progression (25). SMILES notation was used for ligand representation (26, 27), whereas protein primary sequences were encoded by assigning integer labels to the 20 standard amino acids. For input standardization, ligand and protein sequences were organized as two-dimensional matrices. These matrix representations were processed through 2 separate 2D-CNN channels, and the features extracted from each channel were concatenated. The combined features were then fed into a regression module to predict DKBA.

## 2. Objectives

The aim of this study was to investigate whether a lightweight 2D-CNN framework using minimally encoded ligand and protein sequences could achieve useful predictive performance for DKBA. The proposed framework was intended as a computational approach for the preliminary prioritization of candidate compounds targeting kinase proteins, including those potentially relevant to cancer-related research.

## 3. Methods

Figure 1 provides a schematic overview of the proposed DKBA prediction framework. Figure 1A illustrates the preprocessing steps, whereas Figure 1B summarizes the key stages: ligand and protein encoding, feature extraction via two independent convolutional pathways, feature concatenation, and the final regression module for binding-affinity estimation.

**Figure 1.**
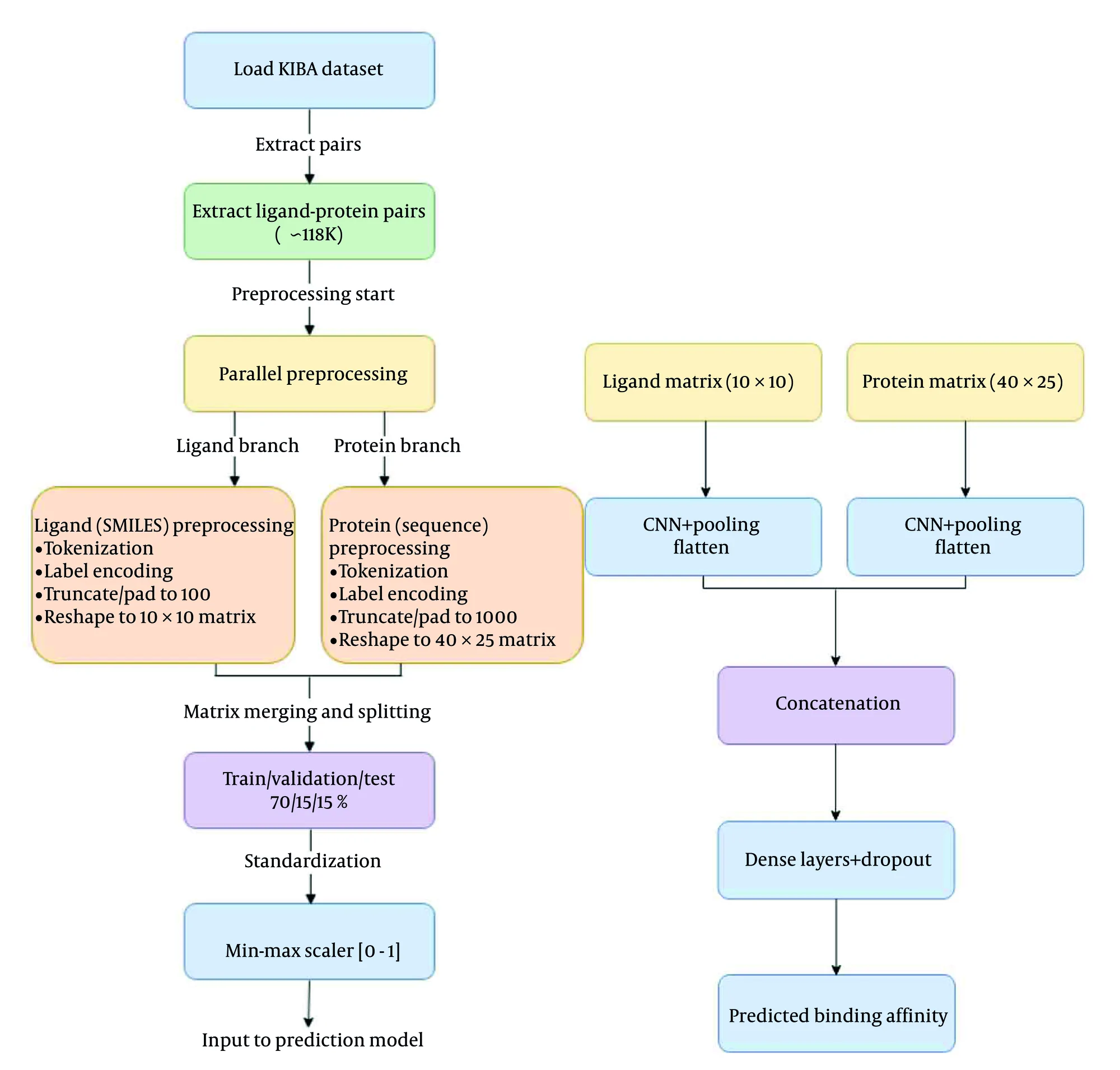
Workflow of the study: A, preprocessing; B, processing flow.

### 3.1. Benchmark Dataset

The publicly available KIBA dataset is a widely used benchmark for DKBA prediction and was specifically developed to study the biological activities of kinase inhibitors. The dataset was originally reported by Tang et al. (28) and combines three key biological indices: dissociation constant (Kd), inhibition constant (Ki), and half-maximal inhibitory concentration (IC50). These indices are integrated into a single KIBA score, which provides a comprehensive measure of the biological activity and binding affinity of drug-kinase protein complexes. The KIBA dataset contains 229 kinase proteins, 2,111 drugs, and 118,254 drug-protein interactions, forming a sparse interaction matrix. KIBA scores range from 0.0 to 17.2, with lower scores indicating stronger binding affinity. The dataset is organized as a drug-protein interaction matrix in which each row represents a drug, each column represents a protein, and each entry represents a KIBA score.

### 3.2. Representation of Model Inputs

This subsection describes the ligand and protein encoding procedures used to prepare and standardize the data for the deep learning model. The data were transformed into formats suitable for identifying and analyzing complex patterns in drug-protein interactions.

#### 3.2.1. Ligand Encoding

SMILES notation was used for ligand encoding. Across the KIBA ligands, 29 unique characters were identified and mapped to integers 1 - 29. The SMILES mapping is provided in Table S1 in the Supplementary File. Among the 2,111 ligand records, 2,068 unique SMILES strings and 2,111 unique ChEMBL identifiers were identified. No invalid or missing SMILES sequences were found. Fifty-seven ligands exceeded 100 characters and were truncated, whereas shorter sequences were zero-padded. The sequences were reshaped into 10 × 10 matrices as a computational design choice to enable 2D convolutional operations rather than to represent molecular geometry or chemical structure.

#### 3.2.2. Protein Encoding

Protein primary sequences were encoded by assigning integers 1 - 20 to the 20 standard amino acids. Among the 229 protein sequences, no duplicate, invalid, or missing sequences were identified. Forty-four protein sequences exceeded 1,000 amino acids and were truncated, whereas shorter sequences were zero-padded. The resulting sequences were reshaped into 40 × 25 matrices as a computational design choice to enable 2D convolutional operations rather than to represent protein structure or biological spatial organization. The amino-acid mapping is provided in Table S2 in the Supplementary File.

### 3.3. Model Architecture

A lightweight dual-channel 2D-CNN model was designed with separate processing pathways for ligands and proteins, followed by feature fusion and regression.

#### 3.3.1. Ligand Processing Pathway

Ligand inputs entered the network as 10 × 10 matrices. First, the inputs were converted into four-dimensional tensors with one channel to enable processing by a 2D-CNN. Three convolutional layers with 3 × 3 kernels and 16, 32, and 64 filters, respectively, were applied. The output was then flattened and passed to a one-dimensional convolutional layer comprising 64 filters with a kernel size of 6. Finally, a global average pooling layer was used to reduce dimensionality and generate a compressed vector of the extracted features.

#### 3.3.2. Protein Processing Pathway

Protein inputs entered the model as 40 × 25 matrices and, similar to the ligand pathway, were converted into four-dimensional tensors with one channel for 2D-CNN processing. This pathway comprised three consecutive convolutional layers with 3 × 3 kernels and 16, 32, and 64 filters, respectively. After the output of these layers was flattened, a one-dimensional convolutional layer with 128 filters and a kernel size of 6 was applied. Finally, a global average pooling layer was used to compress the features.

#### 3.3.3. Feature Fusion and Regression

Feature vectors obtained from the ligand and protein pathways were combined using a concatenation layer and provided as input to the regression module. The regression module consisted of two fully connected (dense) layers with 512 and 64 units, respectively, followed by a single-unit output layer. ReLU activation and L1L2 regularization were used in the hidden dense layers to control model complexity and reduce overfitting. Dropout layers with a rate of 0.5 were applied between the dense layers. The final output layer used linear activation to predict the continuous KIBA affinity score. The detailed model architecture is provided in Table S3 in the Supplementary File.

#### 3.3.4. Training and Optimization

Model training used the Adam optimizer with a learning rate of 0.001 and a batch size of 64, and mean squared error (MSE) was used as the loss function. To reduce overfitting, early stopping was applied with a patience of 20 epochs and a minimum improvement threshold of 0.001. A maximum of 120 training epochs was specified. The L1 and L2 regularization coefficients in the dense layers were set to 10^-6^ and 10^-4^, respectively, and a dropout rate of 0.5 was used. After training and test-set evaluation, the final model was used for DKBA prediction. The model was implemented in Python 3.11.14 using TensorFlow 2.20.0 and Keras 3.13.0 and was trained on an NVIDIA RTX A4500 GPU using CUDA 12.5.1 and cuDNN 9. A fixed random seed of 42 was used to improve reproducibility.

## 4. Results

### 4.1. Evaluation Metrics

MSE, root mean squared error (RMSE), and the concordance index (CI) were used to assess the effectiveness of the proposed model. These metrics are widely used for regression models and provide complementary information on model accuracy and predictive performance.

MSE reflects the mean squared deviation between model predictions and the corresponding true values. RMSE, calculated as the square root of MSE, is expressed on the same scale as the original KIBA scores and is therefore more directly interpretable:

$$\mathrm{MSE}=\frac{1}{n}\sum_{i=1}^{n}{(y_{i}-{\hat{y}}_{i})}^{2}$$

where y_i_ and y ^_i_ denote the actual and predicted affinities for the i-th sample, respectively, and n is the number of samples. MSE also served as the loss function during training.

CI evaluates the model’s ability to correctly rank pairs by affinity:

$$\mathrm{CI}=\frac{1}{Z}\sum_{a_{i}>a_{j}}^{n}\mathrm{h(}p_{i}-p_{j})$$

where p_i_ and p_j_ are the predicted values for pairs with actual affinities a_i_ > a_j_, Z is a normalization constant, and h(x) is the step function:

$$h\left(x\right)=\left\{ \begin{array}{ccc} 1 & \text{for} & x>0\\ 0.5 & \text{for} & x=0\\ 0 & \text{for} & x<0 \end{array}\right.$$

### 4.2. Model Training and Validation Strategy

The KIBA interaction pairs were randomly shuffled and split into training (70%), validation (15%), and test (15%) sets. Thus, splitting was performed at the drug–protein interaction-pair level rather than by grouping drugs or proteins. Consequently, the same drug or protein could appear in more than 1 subset, whereas each specific drug–protein interaction pair was assigned to only 1 subset.

For label preprocessing, Min-Max scaling was fitted exclusively to the training-set KIBA scores. The resulting scaling parameters were then applied to the validation set without refitting. During model training, the scaled training and validation labels were used for optimization. After prediction on the test set, the predicted values were transformed back to the original KIBA scale using the inverse transformation based solely on the training-set scaling parameters. All reported test-set performance metrics were then computed using the back-transformed predictions and the original, unscaled KIBA test-set scores. Therefore, no information from the validation or test labels was used to fit the scaling transformation.

The MSE loss function was used to measure prediction error during training and validation. The model was trained using the Adam optimizer with a learning rate of 0.001 and a batch size of 64. To reduce overfitting, early stopping was applied with a patience of 20 epochs and a minimum improvement threshold of 0.001. Figure 2 illustrates the training and validation MSE curves during training. The training and validation losses showed similar decreasing trends, suggesting stable optimization without evident overfitting.

**Figure 2.**
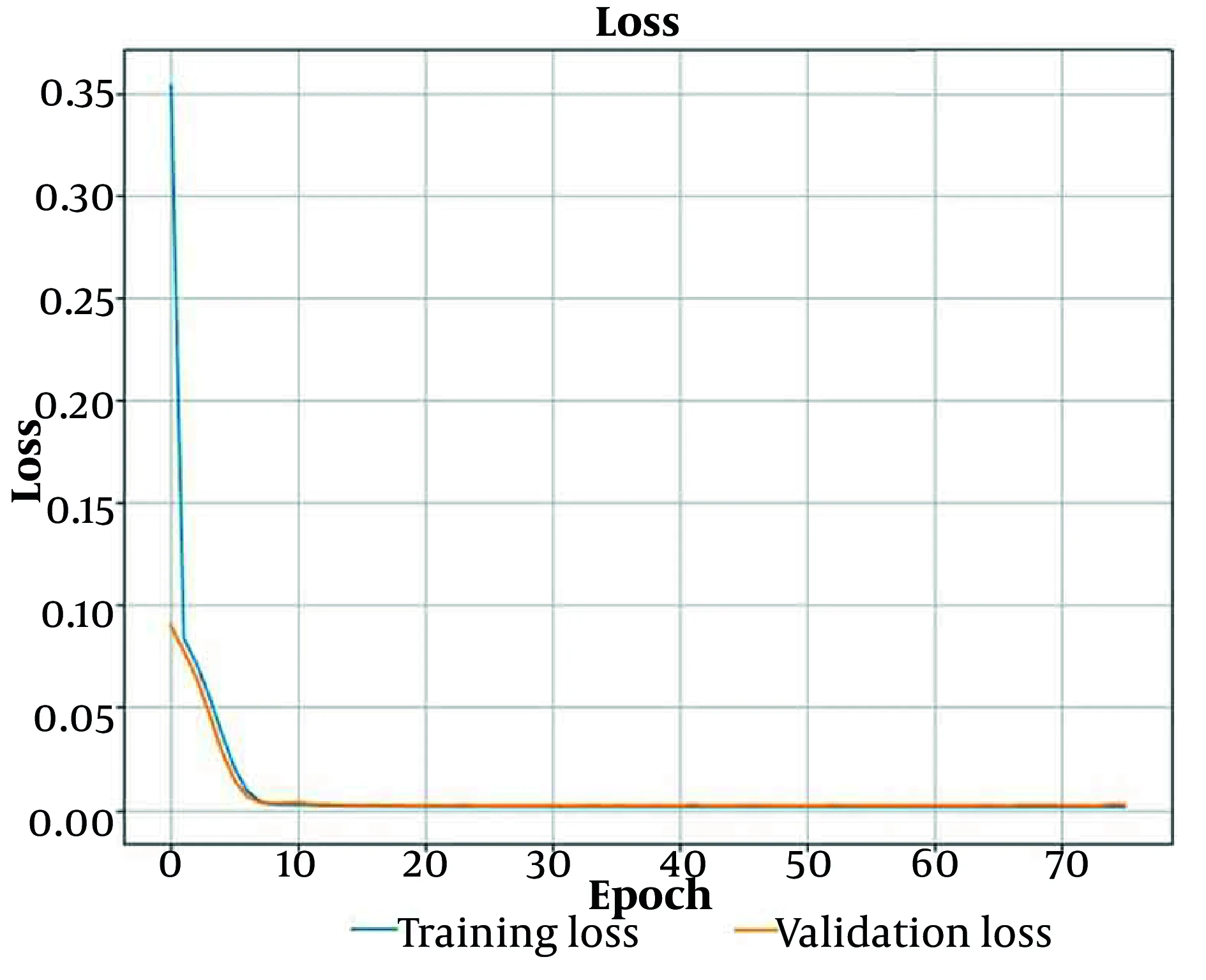
Evolution of mean squared error across training epochs.

### 4.3. Model Performance on the Test Set

This subsection presents the performance metrics (MSE, RMSE, and CI) of the proposed model and literature-reported results for baseline and standard models for DKBA prediction. All performance metrics for the proposed model were calculated using the original, unscaled KIBA scores of the test set. The performance values reported for SimBoost, KronRLS, DeepDTA, and WideDTA were obtained from their original publications and were not reproduced using the preprocessing pipeline of the present study. Therefore, these values are presented as literature-reported reference results and should not be interpreted as strictly controlled head-to-head comparisons. Table 1 summarizes the results; lower MSE and RMSE values and a higher CI indicate better performance. RMSE was additionally reported to provide an error measure on the original KIBA-score scale.

**Table 1.** Performance Metrics of the Proposed Model and Literature-Reported Results for Baseline (SimBoost and KronRLS) and Standard Deep Learning Models (DeepDTA and WideDTA) on the KIBA Dataset

Model	MSE	RMSE	CI	Source
**The proposed model**	0.184	0.429	0.870	This study
**SimBoost**	0.222	0.471	0.836	Ref. 16
**KronRLS**	0.411	0.641	0.782	Ref. 15
**DeepDTA**	0.194	0.440	0.863	Ref. 17
**WideDTA**	0.179	0.423	0.875	Ref. 18

The proposed model achieved lower MSE and RMSE values and a higher CI than the literature-reported results for SimBoost and KronRLS, whereas its performance was close to that reported for DeepDTA and WideDTA. However, because these reference models were not reproduced using the same data split and preprocessing pipeline, these differences should be interpreted as contextual comparisons rather than direct evidence of superior performance. Despite its relatively simple input representation, the proposed 2D-CNN achieved acceptable predictive performance on the KIBA test set. This finding suggests that a lightweight architecture can provide useful predictive performance with a relatively simple representation, although its potential application in large-scale or early-stage virtual screening requires further validation under controlled and independent evaluation settings.

Figure 3 shows the correspondence between the model-estimated and true values for the test data. The horizontal axis represents the ground-truth affinities, and the vertical axis represents the model-predicted values. As expected, closer alignment of points around the diagonal line reflects better predictive performance. The scatter plot indicates that most test data points are reasonably distributed around this line, suggesting acceptable prediction accuracy for the proposed model.

**Figure 3.**
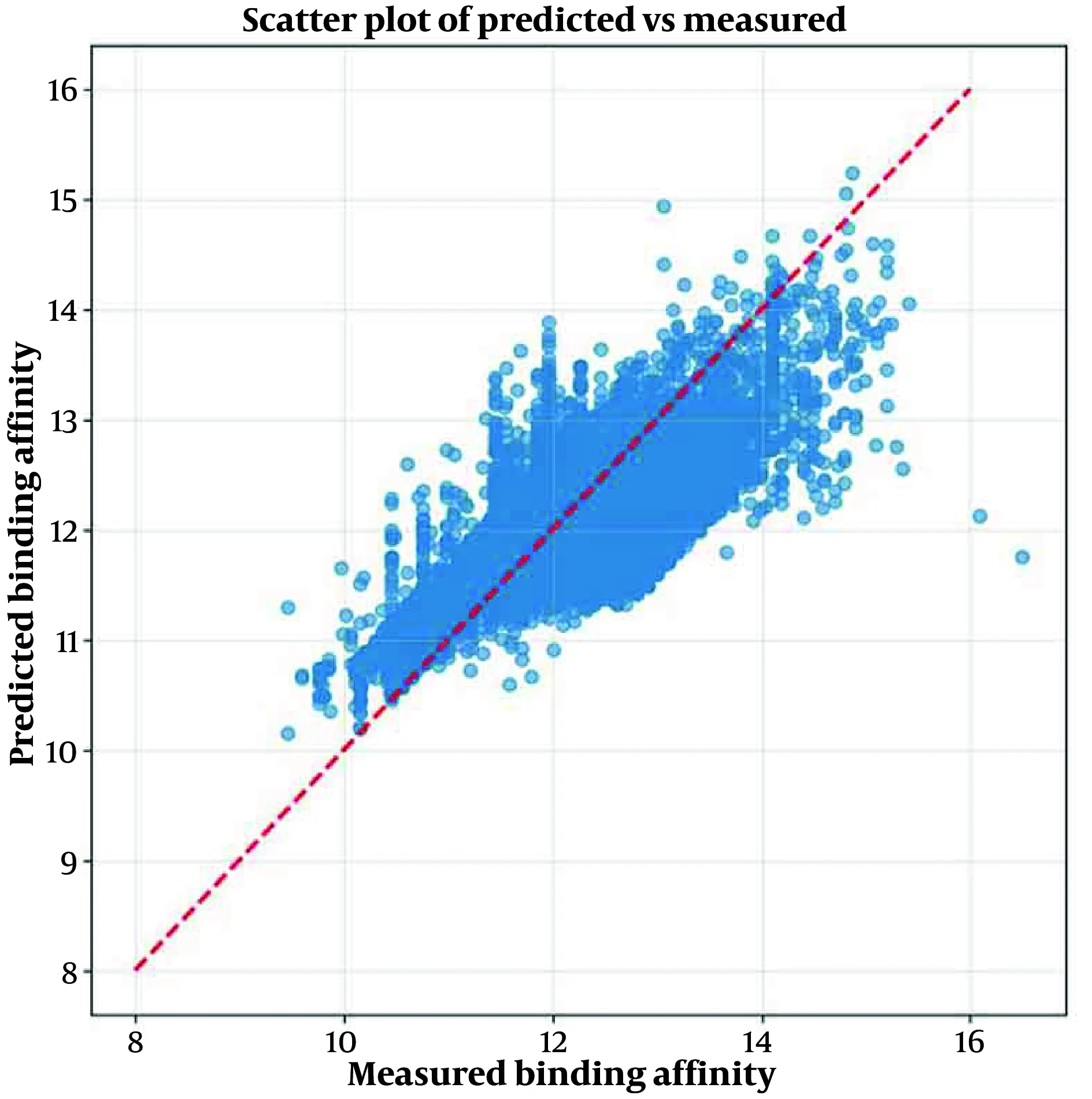
Predicted vs measured binding affinity on the KIBA test set.

### 4.4. Model Complexity and Computational Efficiency

The computational characteristics of the proposed model were quantified to support the lightweight design objective. The model contained 179,649 trainable parameters, corresponding to approximately 0.685 MB of parameter storage assuming 32-bit floating-point representation. Training on an NVIDIA RTX A4500 GPU required approximately 56 minutes over 31 epochs before early stopping. In addition, the mean inference time was approximately 0.597 ms per sample, corresponding to a throughput of approximately 1,674 samples per second.

## 5. Discussion

The proposed model achieved acceptable predictive performance on the KIBA test set using straightforward label-encoded ligand and protein sequences. Its performance was numerically close to the literature-reported results of established deep learning approaches such as DeepDTA and WideDTA, whereas lower MSE and RMSE values and a higher CI were observed relative to the reported results for SimBoost and KronRLS. However, because these benchmark results were obtained from the original publications rather than reproduced using the same data split and preprocessing pipeline, these numerical differences should be interpreted as contextual rather than as direct evidence of superior performance.

A key insight from this work is that CNNs can detect latent ligand-protein interaction patterns even with basic label encoding of SMILES strings and amino acid sequences. This finding suggests that CNN architectures can learn useful patterns from matrix-like representations without complex graph representations, attention mechanisms, or pretrained embeddings.

Compared with graph-based models that use molecular structures, the proposed architecture offers a simpler and more computationally efficient approach with minimal preprocessing. To the best of our knowledge, this study provides an empirical evaluation of a lightweight 2D-CNN architecture for DKBA prediction using minimally encoded ligand and protein sequences without requiring graph-based encoders, attention mechanisms, or pretrained embeddings.

Another important observation is that the model maintained stable training and validation performance during optimization. The similarity between the training and validation losses, together with the use of regularization, dropout, and early stopping, suggests that overfitting was not observed under the predefined random interaction-level split. These findings support the potential of the proposed CNN architecture for DKBA prediction using a simple encoded representation. However, this evaluation does not establish generalization to completely unseen drugs or proteins, and more stringent drug-disjoint, protein-disjoint, or scaffold-based validation would be required to assess such generalization.

It is important to emphasize that AI-based ligand screening typically serves as an initial step in an in silico drug-discovery pipeline. The primary objective of this stage is to prioritize promising compounds rather than to achieve perfect prediction accuracy. To validate compounds selected during the AI stage, prioritized ligands should subsequently be re-evaluated using structure-based approaches such as molecular docking and molecular dynamics simulations. Therefore, an early-stage prediction model does not necessarily require extremely high accuracy but should be fast, scalable, and able to reliably prioritize candidates for downstream structure-based evaluation.

### 5.1. Study Limitations

The primary limitation of the proposed framework is the simplicity of the input representation. Integer label encoding assigns numerical values to categorical SMILES characters and amino acids and therefore does not explicitly preserve chemical or biological similarity between different tokens. Although the CNN was able to learn useful patterns from these representations, richer encoding strategies, such as physicochemical feature vectors, pretrained embeddings, or structure-aware molecular representations, may capture additional chemical and biological information. However, incorporating such representations would generally increase preprocessing and computational complexity, representing a trade-off between representational richness and the simplicity and efficiency targeted by the present lightweight framework. In addition, truncating sequences that exceed the selected limits may result in some information loss. Nevertheless, only 57 of 2,111 ligands and 44 of 229 proteins exceeded the selected limits. The selected thresholds provided a practical balance among truncation, excessive zero-padding, and input size. Furthermore, the model was not evaluated on independent or disease-specific datasets, which limits assessment of its generalizability to practical drug-discovery applications.

### 5.2. Conclusions

In summary, this study presents a simple yet efficient framework for predicting drug-kinase protein binding affinity using encoded ligand and protein sequences. Although there is room for improvement, the proposed model lays the groundwork for further research aimed at enhancing the interpretability and predictive robustness of deep learning-based approaches in computational drug discovery.

This study presents a lightweight 2D-CNN-based framework for predicting DKBA using label-encoded ligand and protein sequences. The model achieved acceptable predictive performance on the KIBA test set despite using a simple input representation and minimal preprocessing. The results suggest that such a lightweight architecture may provide a useful foundation for computational DKBA prediction. However, the present evaluation does not establish superiority over existing methods because the benchmark results were obtained from the literature rather than reproduced under identical experimental conditions. Moreover, external validation and disease-specific evaluation were not performed. Future studies should therefore investigate more stringent validation strategies, improved sequence representations, and independent disease-specific datasets to further assess the generalizability and practical utility of the proposed approach.

ijpr-25-1-172781-s001.pdf

## Data Availability

The data is open source and available freely on the web. The source code can be provided to interested researchers upon reasonable request.
