# Supplementary material for: Lightweight 2D-Convolutional Neural Network for Drug-Kinase Binding Affinity Prediction with Minimal Encoded Representations
Source: Iran J Pharm Res. 2026 Sep 26;25(1):e172781. doi: 10.5812/ijpr-172781 (PMC13647229; doi:10.5812/ijpr-172781)
Supplement: Supplementary file 1 [file ijpr-25-1-172781-s001.pdf]

Supplementary Table S1. Character vocabularies and integer mappings used for SMILES sequences

Character	Integer label
c	1
=	2
(	3
)	4
n	5
1	6
2	7
o	8
3	9
4	10
f	11
5	12
s	13
l	14
6	15
#	16
7	17
b	18
r	19
[	20
]	21
8	22
+	23
-	24
i	25
9	26
.	27
p	28
h	29

Supplementary Table S2. Character vocabularies and integer mappings used for protein sequences

Character	Integer label
l	1
s	2
e	3
k	4
g	5
a	6
v	7
r	8
p	9
d	10
t	11
i	12
q	13
f	14
n	15
y	16
h	17
m	18
c	19
w	20
